# Public health surveillance of *Yersinia* species in England, 2017–2024

**DOI:** 10.1017/S0950268826102167

**Published:** 2026-10-14

**Authors:** Ella V. Rodwell, Ella te Wierik Durrant, Nicola K. Love, David R. Greig, Grace King, Iain Hayden, Vanessa Wong, Marie Anne Chattaway, Claire Jenkins, Gauri Godbole

**Affiliations:** 1 UK Health Security Agency, UK; 2 Health Protection Research Unit in Gastrointestinal Infections, National Institute for Health and Care Research, UK; 3 Health Protection Research Unit in Genomics and Enabling Data, National Institute for Health and Care Research, UK

**Keywords:** Epidemiology, Yersinia enterocolitica, Yersinia pseudotuberculosis, Gastrointestinal infection, Surveillance

## Abstract

*Yersinia pseudotuberculosis* and *Yersinia enterocolitica* are globally found zoonotic gastrointestinal bacteria known to cause human infections, transmitted by consumption of contaminated food or environmental contact. *Y. enterocolitica* is the dominant species in causing yersiniosis, and is under ascertained in England. Diagnostic laboratories across England undertake detection of *Yersinia* in stool and other sterile sites using UK Standards for Microbiology Investigations. In 2022, there was an exceedance of invasive and gastrointestinal *Yersinia* notifications across England. Recommendations were made that all *Yersinia* detected in diagnostic laboratories be cultured and sent to England’s national reference laboratory, Gastrointestinal Bacteria Reference Unit, UK Health Security Agency for whole genome sequencing, speciation, genomic profiling, and clustering. Here we describe the epidemiology of all *Yersinia* spp. reported in England to the UKHSA from the regional laboratories and the reference laboratory leading up to and following the exceedance in 2022. We aim to capture and describe the changes and developments in surveillance of *Yersinia* spp. between 2017 and 2024.

## Introduction

*Yersinia* is a diverse genus comprising at least 28 different species, the majority of which are found in animals and/or the environment and are not known to cause clinical symptoms in humans [1–7]. There are three well-established pathogenic species: *Yersinia pestis, Yersinia pseudotuberculosis*, and *Yersinia enterocolitica.* The focus of this surveillance is primarily gastrointestinal yersiniosis; therefore *Y. pestis*, the causative agent of bubonic, pneumonic, and septicemic plague is not included [8, 9]. This study will also report on *Yersinia* spp. not previously associated with gastroenteritis but have been detected in specimens provided by cases that presented to healthcare with gastroenteritis-like symptoms.

*Y. pseudotuberculosis* and *Y. enterocolitica* are zoonotic gastrointestinal (GI) bacterial pathogens transmitted to humans through contact with animals and their environment and/or consumption of contaminated food [10]. Raw or undercooked pork products and unpasteurized dairy products are considered high-risk foods, and more recently fresh produce contaminated with animal faeces has also been implicated [10, 11]. Although pigs are the main reservoir for *Y. enterocolitica*, both species have been isolated from other farm and domesticated animals, such as poultry, cattle, sheep, goats, cats, dogs, and from wild animals, for example rodents, deer, and boars, as well as being ubiquitous in the environment [12–14].

Although *Y. pseudotuberculosis* is less common in humans compared to *Y. enterocolitica*, both *Y. enterocolitica* and *Y. pseudotuberculosis* are known to cause an enteric illness called yersiniosis, presenting with self-limiting gastroenteritis (diarrhoea with or without blood, cramping, sickness) which often resolves in 2–3 weeks [15, 16]. *Yersinia* can move extra-intestinally and cause invasive infections such as bacteraemia, with both *Y. enterocolitica* (biotype 1B more often) and *Y. pseudotuberculosis* reported to cause invasive infection [17–19]. Serious complications can develop from yersiniosis including ileitis, appendicitis, and sepsis, with secondary immunological complications presenting as reactive arthritis, Reiter’s disease and erythema nodosum also possible. Antibiotics are recommended in severe cases, with trimethoprim-sulfamethoxazole, aminoglycosides, third-generation cephalosporins, fluoroquinolones, and tetracyclines showing susceptibility in *Y. enterocolitica* [15, 20, 21].

*Y. enterocolitica* is the fourth most reported GI pathogen in Europe [10], with a notification rate in 2022 of 2.2 cases per 100000 compared to European *Campylobacter* and *Salmonella* reports in 2022: 46.9 and 15.5 cases per 100000 respectively [22, 23]. The highest rates were seen in the 0–4 age group at 10.1 cases per 100000 [10]. Comparatively, England and the UK recent rates were around 0.2–0.3 cases per 100000 between 2016 and 2020 [10, 24]. This is considerably lower than in European countries and is likely due to under-ascertainment rather than a true reflection of incidence. The under-ascertainment in England is predicated to be ~7500 cases/year [24]. This is equivalent to 13.2 per 100000 infections using 2020 mid-year estimates. The UK Standards for Microbiology Investigations (UK SMIs) do not recommend screening all faecal specimens from cases of gastroenteritis for *Yersinia* spp. [25]. Current guidelines state ‘Culture for this organism is only recommended when clinical presentation of appendicitis, mesenteric lymphadenitis/adenitis, terminal ileitis, reactive arthritis is associated with gastroenteritis and in cases of unresolved gastroenteritis where *Salmonella* species and *Campylobacter* species have been ruled out, particularly when associated with travel outside the UK’ [25, 26].

Although culture of *Yersinia* spp. is challenging, it can be performed using selective cefsulodin-irgasan-novobiocin (CIN) culture media for 24–48 h at 28°C–30°C on samples from stool, or other relevant extraintestinal sites depending on the symptoms (e.g. blood, bile, mesenteric lymph node, cerebrospinal fluid, or peritoneal fluid) [27]. Commercial GI Polymerase Chain Reaction (PCR) assays provide the potential for testing faecal specimens for a wider range of targets. Where laboratories have implemented PCR methods for detection of *Y. enterocolitica*, higher case rates have been reported [24]. Testing algorithms for GI pathogens vary between clinical laboratories, and it is not uncommon for *Yersinia* culture to be restricted to cases where a relevant travel history has been reported. Given the generally low rates reported across England, individual laboratories using PCR for *Yersinia* detection have a noticeable impact on the surveillance data and provide evidence of under-ascertainment in England.

In spring 2022, a national exceedance in *Yersinia* spp. was detected in England, and recommendations were made that all *Yersinia* detected in diagnostic laboratories be cultured and sent to England’s national reference laboratory, Gastrointestinal Bacteria Reference Unit (GBRU), UK Health Security Agency (UKHSA) (formerly Public Health England (PHE)). Referrals to the GBRU were requested to continue after the 2022 exceedance period for confirmatory testing and whole genome sequencing (WGS).

Here we describe the epidemiology of *Yersinia* spp. reported in England to the UKHSA from various available data sources, leading up to and following the exceedance in 2022. We aim to capture and describe the changes and developments in surveillance of *Yersinia* spp. between 2017 and 2024.

## Methods

### Diagnostic laboratory detection and reporting

Diagnostic laboratories across England undertake detection of *Yersinia* in stool and other sterile sites using UK SMIs [25], though some laboratories may restrict testing to cases with reported travel or where there is a clinical indication such as appendicitis-like illness or mesenteric adenitis [28]. Sterile specimen sites are defined as being obtained from body sites that are typically free of microorganisms in healthy conditions (sample types include blood, cerebrospinal fluid). Non-sterile specimen sites were collected from sites with natural microbial flora (sample types include stool, sputum, mouth, skin).

Although non-pestis *Yersinia* was not a mandatory notifiable infection in England when this surveillance was undertaken [29], most diagnostic laboratories in England have been reporting detection of *Yersinia* spp. to the UKHSA via the Second Generation Surveillance System (SGSS). However, there is variation across laboratories with testing and reporting of *Yersinia.* Some laboratories will report *Yersinia* to UKHSA if identified by PCR, whereas others will only report *Yersinia* once a PCR positive is confirmed by culture. There is variation in the commercial enteric PCR panels used in diagnostic laboratories (including BioFire, SeroSep and Launch Diagnostics), some of which detect *Y. enterocolitica* and others *Yersinia* spp. Information on patient demographics (age, sex, region of residence) and specimen details (specimen date and type, test method, laboratory, organism, and species) for *Yersinia* cases reported between 2017 and 2024 were extracted. Records were deduplicated using available demographic data into episodes of 28-days, retaining the first sample following deduplication. Cases with records indicating detections of more than one *Yersinia* species were deduplicated, retaining the first sample if specimen results were reported within 28 days.

### Reference laboratory detection and reporting

All isolates referred to the GBRU undergo whole genome sequencing (WGS) and characterization as methods described previously [30, 31]. Briefly, Genomic DNA was extracted, fragmented, and tagged for multiplexing with Nextera XT DNA Sample or Nextera DNA Flex Preparation kits (Illumina) and sequenced using the Illumina HiSeq 2500 and NextSeq 1000 platforms at UKHSA. Reads were trimmed using Trimmomatic (v0.32). The FASTQs produced during WGS were used to identify *Yersinia* species using KmerID, a validated Kmer-based approach used in UKHSA pipelines (https://github.com/ukhsa-collaboration/kmerid, [30, 32–35]). The FASTQ reads from all sequences in this study can be found at the UKHSA Pathogens (*Yersinia*) BioProject at the National Center for Biotechnology Information (PRJNA481015).

Since 5 January 2023, all diagnostic laboratories in England were requested to submit *Yersinia* isolates to the GBRU for confirmation of species and typing. The reference laboratory results are stored in the Gastro Data Warehouse (GDW), linking WGS, and patient information such as age, sex, and recent travel included in the laboratory request form. GDW records between 01 January 2017 and 31 December 2024 were extracted. Cases were deduplicated using available demographic data into episodes of 28-days, retaining the first sample following deduplication.

Case definitions were harmonized across the two datasets using patient-identifiable information and 28 days to identify cases. This study is surveillance and notification of *Yersinia*, rather than detailed strain resolution or investigation of potential dual detections identified through WGS. Therefore, dual detections with multiple *Yersinia* species within a 28-day window were deduplicated to the first record for incidence purposes (with an exception when presenting dual invasive detections as this presents clinical relevance, indicated by ^‡^). This is due to the inability to speciate *Yersinia* at the regional laboratories and for the purpose of harmonizing case definitions. Further analysis into dual detections will be investigated outside of this study.

Antimicrobial susceptibility was not routinely undertaken or reported in England due to lack of standardized methods (EUCAST criteria) for non-pestis *Yersinia* spp. during the period of this study. To begin to understand antimicrobial susceptibility in *Yersinia*, a random selection of pure isolates of *Y. enterocolitica* and *Y. pseudotuberculosis* from stool and blood between 2017 and 2023 were tested for antimicrobial susceptibility using microbroth dilution [36]. The minimum inhibitory concentrations (MIC) were recorded for standardized panel of antibiotics (Sensititre EUVSEC2 plate and EUVSEC3 [37, 38]) and resistance was inferred using the EUCAST breakpoint table for *Enterobacterales* [39].

### National survey of laboratory detection of enteric pathogens in England

In 2023, a survey was undertaken in England to map the various methods used by diagnostic laboratories for detection of various GI pathogens. An electronic self-complete questionnaire was developed and distributed to laboratories in England. The questionnaire requested testing methods, geographic data, and diagnostic capabilities available for GI pathogens. Targeted questions were asked on community populations tested, methods of detection, identification, and antimicrobial sensitivity for the *Yersinia* (and other GI pathogens) detected from faeces.

### Epidemiological analysis

For general trend analysis, case records identified in SGSS and GDW were not linked with each dataset and instead were deduplicated using the same criteria and analysed separately with the counts and trends compared. Records were split into two time points, between 2017–2021 and 2022–2024. There are variations in referrals to the reference laboratory, as well as bidirectional data gaps between SGSS and GDW records. If data were linked between the two sources and only matched data were taken forward, the study would have an unrepresentative case load (~40%) for trends, and conclusions could not be drawn.

For sample type investigations, cases between SGSS and GDW were matched on full name and date of birth. This is to ensure that independent sample types do not influence conclusions drawn; however, there are limitations on data matching based on spelling and middle name presence.

Rates of detection per 100000 population per year were calculated by sex, age group (0–4, 5–14, 15–24, 25–44, 45–64, >65) [40], and region [41] using data from ONS mid-year population estimates [40]. Mid-year estimates for 2023 were used for 2024 as more recent estimates were not available. Detections were calculated per year and across the two time points (2017–2021 and 2022–2024). Mean population and cases were used to calculate the detection per 100000 persons across the two time points.

## Results

### Rate of detections and regional reporting

**Figure 1.**
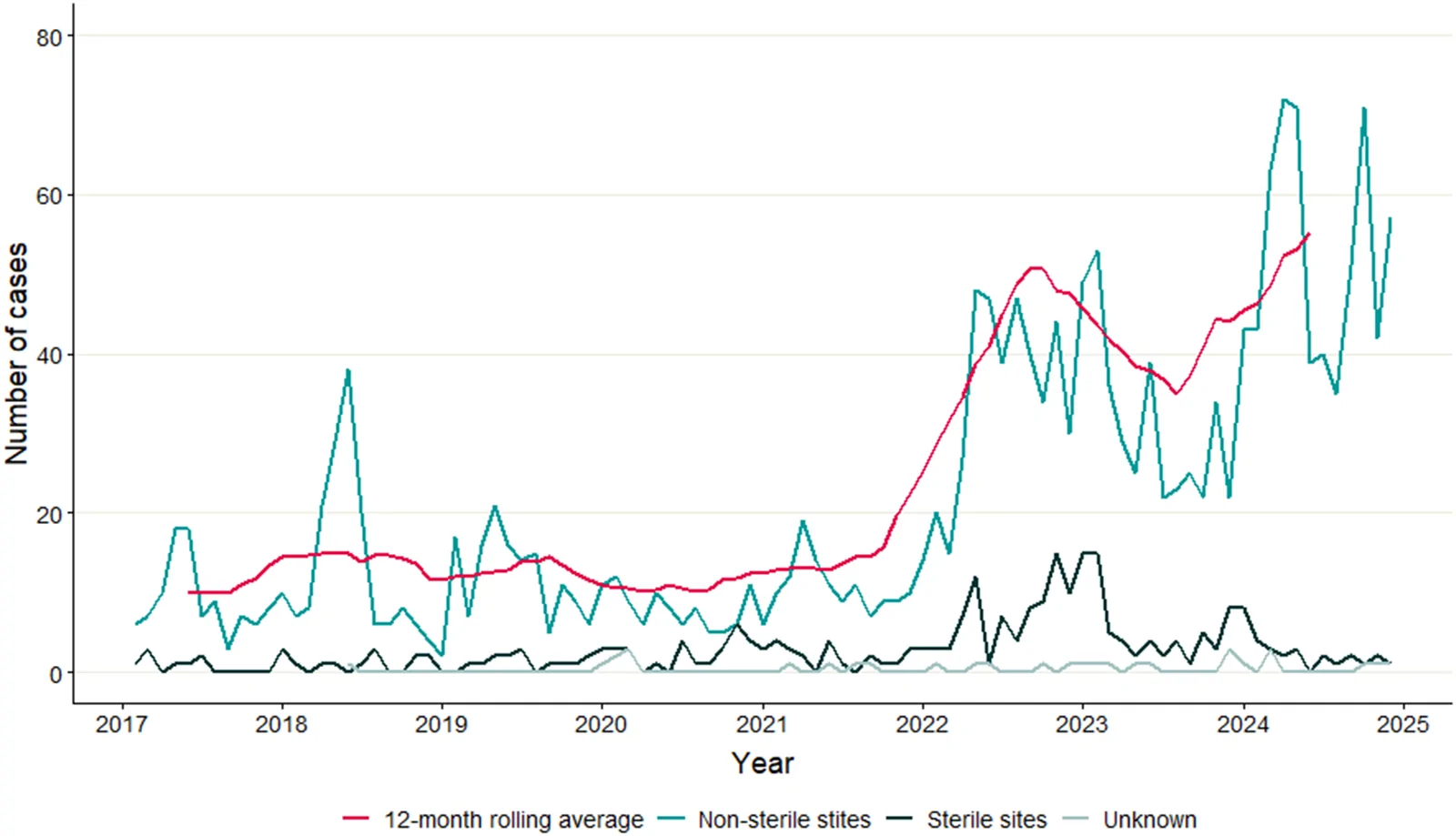
*Yersinia* cases reported to SGSS in England. Case defined as <28 day episode. Case data between 2017 and 2024 are portrayed longitudinally to identify seasonal and yearly trends. Light blue line indicates monthly case load of non-sterile sites, teal indicates monthly case load of sterile sites, blue-grey indicates monthly case load of those with unknown sites, and pink indicates 12-month rolling average of all cases. Figure 1. long description.

There were 740 detections of *Yersinia* from 733 patients reported by diagnostic laboratories in England between January 2017 and December 2021. Prior to April 2022 the number of cases reported varied from 2 to 39 per month, with seasonal peaks commonly occurring in the summer months (Figure 1).

**Figure 2.**
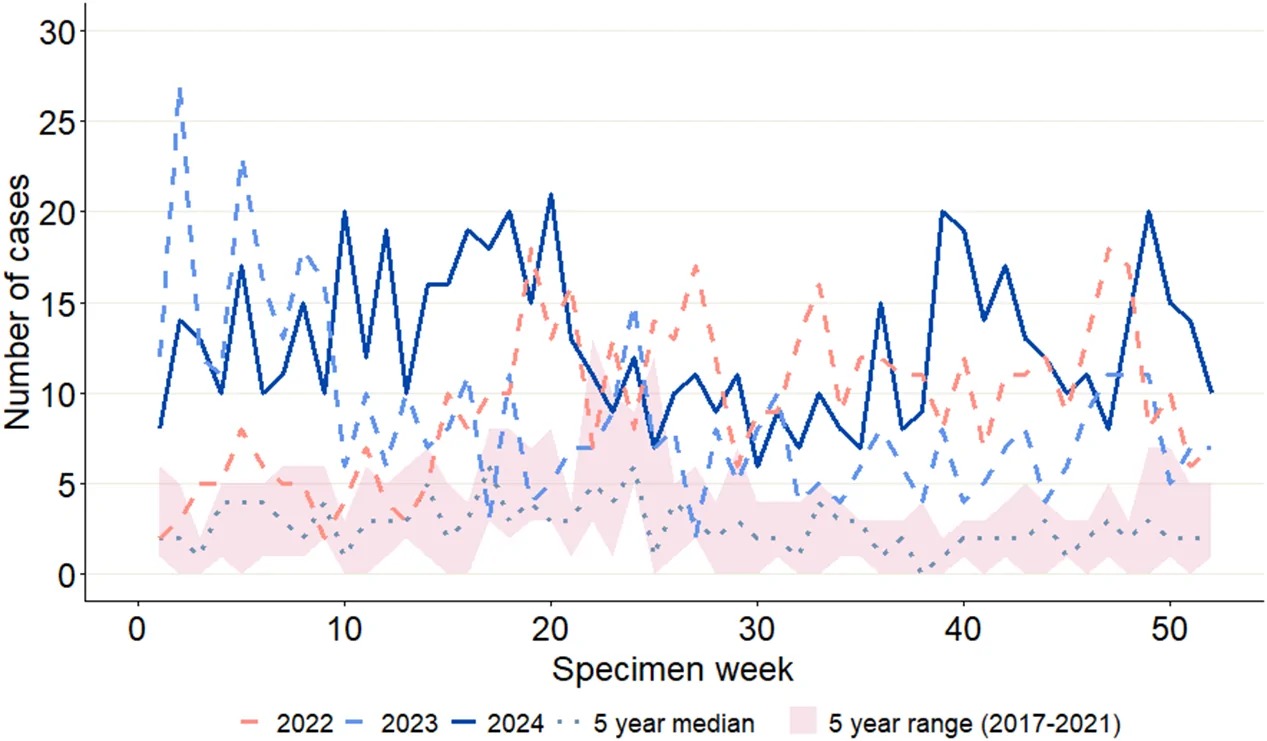
*Yersinia* cases reported to SGSS in England, with 5-year trends overlayed. Case defined as <28-day episodes. Data between 2017 and 2021 were used to calculate the 5-year trend data (maximum and minimum displayed as a pink ribbon with the median dotted. The years 2022 and 2023 are presented as dashed (pink and blue respectively) lines and 2024 solid blue line. Figure 2. long description.

In April and May 2022, a national exceedance occurred, with the weekly cases peaking at week 19 (*n* = 18), an increase of ~4 times above the 5-year mean for that time point (Figure 2). A higher weekly case count was observed throughout 2022 and early 2023 (when compared to 2017–2021) (maximum *n* = 27, week 2, 2023) before reducing in April 2023. Weekly case rates increased again between November 2023 and January 2024 and to date have remained higher than the pre-exceedance period.

Since January 2022, detections of *Yersinia* (*n* = 1611 from 1599 patients) have been significantly higher than 2017–2021 and have remained higher than the pre-exceedance 5-year trends (Welch two-sample t-test of 5-year mean and 3-year mean, *p* < 0.05 (Supplementary Table 1 and Figure 2)). Detections per 100000 population were consistently between 0.2 and 0.3 between 2017 and 2021 (Supplementary Table 1). This increased between 2022 and 2024 (0.9, 0.8, and 1.2 per 100000 respectively) (Supplementary Table 1). It is important to note that some values may differ from previously reported *Yersinia* spp. notifications due to the nature of using a live database and retrospective reporting from laboratories [10, 42].

Regionally, the highest proportion of *Yersinia* detections are those residents in the South East, although this decreased between 2017–2021 and 2022–2024 from 57% to 51%. The second-highest proportion is residents in the North West, increasing from 9% to 17% between 2017–2021 and 2022–2024. A proportional increase was also seen in the South West (6% in 2017–2021 to 9% in 2022–2024) whilst other regions largely remained constant or saw small proportional decreases.

There were 19 patients who had multiple detections of *Yersinia* outside of the 28-day window. The interquartile range of these cases was 97 days [35–132], with a range of 29–1889 days and a median of 64 days. The multiple detections could result from long-term persistence of *Yersinia* in the body, or repeated acquisition or detection. Due to this, the patients’ age at each detection timepoint were counted in the age-sex analysis.

**Figure 3.**
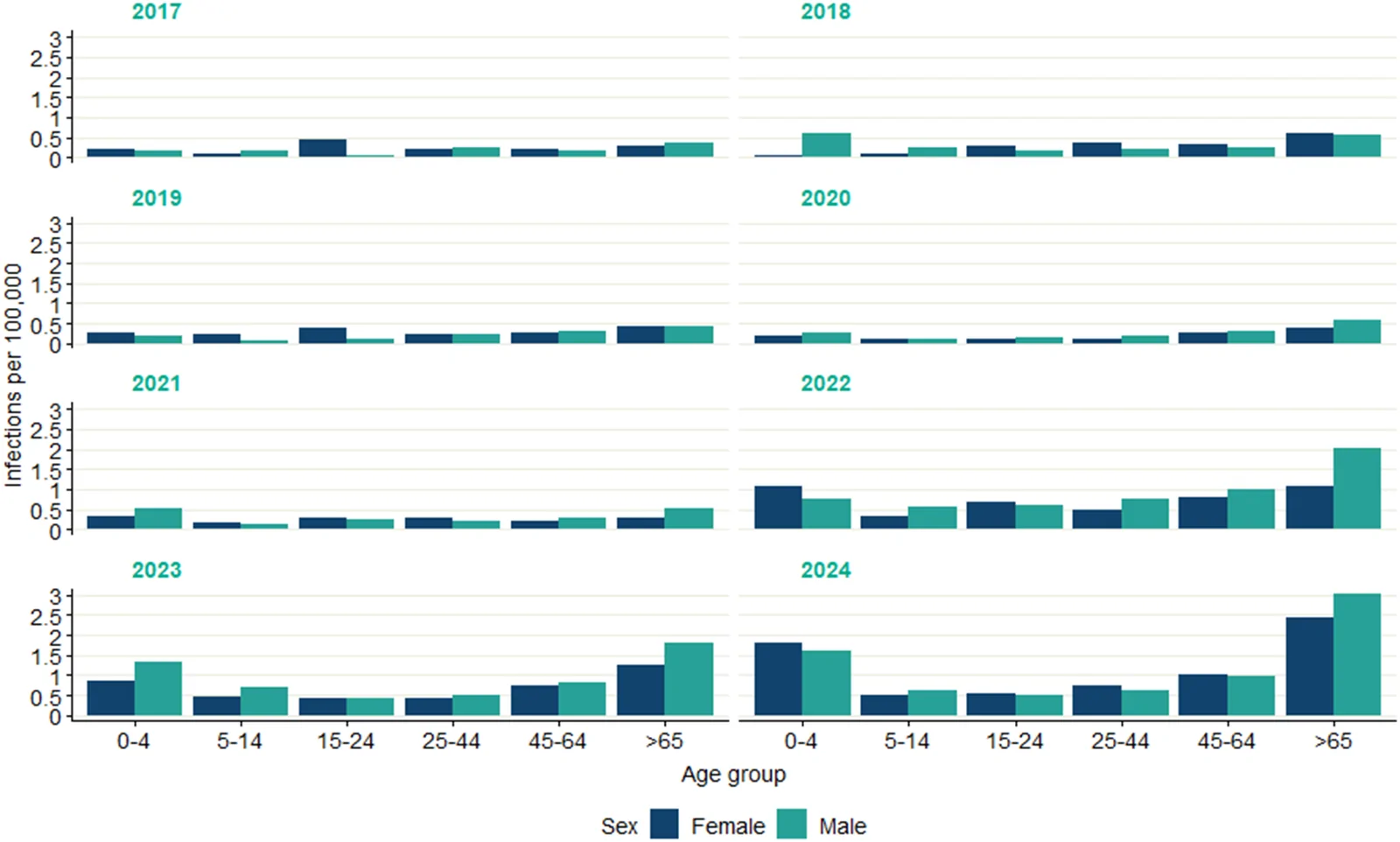
Infections per 100000 per age-sex grouping per year of *Yersinia*. Where age and sex were available for *Yersinia* spp. notified to SGSS (*n* = 2343) and using populations for sex per year were aggregated into the above age groups [40] and used to calculate infections per 100000 persons.

The age-sex distribution of *Yersinia* cases in SGSS shows the highest rate of detection in those above 65 years of age (Figure 3), followed by those below 5 years of age. When the cases were split into pre and post 2022, detections in those above 65 years increase from a percentage proportion of 30% in 2017–2021 to 38% in 2022–2024. The 25–44 age group reduced from 22% to 17%, and the 15–24 age group reduced from 10% to 7%. The remainder of the detection proportions within the other age groups remained consistent between 2017–2021 and 2022–2024 (Table 1). Detections per 100000 population for the above 65 years of age have increased from ~0.3 in 2017 to ~2.8 in 2024 (Figure 3). Additionally, the 0–4 age group detections per 100000 have increased from ~0.18 in 2017 to ~1.7 (Figure 3).

**Table 1.** People, place, and time notifications of yersiniosis from SGSS Table 1. long description.

		2017–2021			2022–2024			Total		
		*n*	%	DpH	*n*	%	DpH	*n*	%	DpH
Age group	0–4	45	6	0.28	116	7	1.26	161	7	0.64
	5–14	48	6	0.14	110	7	0.53	158	7	0.29
	15–24	72	10	0.22	107	7	0.52	179	8	0.33
	25–44	166	22	0.22	275	17	0.59	441	19	0.36
	45–64	188	25	0.26	387	24	0.88	575	24	0.50
	>65	221	30	0.43	615	38	1.91	836	36	1.00
	Total	740	100	NA	1610	100	NA	2350	100	NA
Region	East Midlands	20	3	0.08	43	3	0.29	63	3	0.16
	East of England	38	5	0.12	47	3	0.24	85	4	0.17
	London	65	9	0.15	78	5	0.29	143	6	0.20
	North East	20	3	0.15	71	4	0.88	91	4	0.43
	North West	67	9	0.18	268	17	1.18	335	14	0.56
	South East	425	57	0.92	824	51	2.91	1249	53	1.68
	South West	46	6	0.16	142	9	0.82	188	8	0.41
	West Midlands	23	3	0.08	51	3	0.28	74	3	0.16
	Yorkshire and The Humber	36	5	0.13	87	5	0.52	123	5	0.28
	Total	740	100	NA	1611	100	NA	2351	100	NA

*Note*: Age-sex denominator is *n* = 1610 for 2022–2024 and *n* = 2350 for the totals as one age was missing.

The temporal aspect is split into two groupings, 2017–2021 (considered pre-exceedance) and 2022–2024 (considered during/post exceedance). DpH = Detections per 100000. Average cases across the two time periods for age groups were used against the average population (mid-year estimates across the two time points) to calculate the detection rate per 100000 per age group. 2024 population calculations used 2023 numbers as the mid-year prediction was not available. For age groups [40]. For regions [41]. Some percentages may not sum to 100% due to rounding.

In diagnostic laboratories, the test methods most often reported to SGSS were culture (1157/2351, 49%) and PCR (909/2351, 38%). In addition to PCR, there were 285/2351 (12%) other/unknown methods reported to SGSS (Supplementary Figure 1). Between 2017 and 2021 there were 8 laboratories that reported a PCR record to SGSS, whereas between 2022 and 2024 there were 18 laboratories that reported a PCR record to SGSS. Multiple Laboratory Information Management Systems (LIMS) are used across laboratories to report records to SGSS. Testing and reporting algorithms vary between laboratories reporting to SGSS, with some using both PCR and culture whilst others using just one method. As negative results are not reported to SGSS, and not all laboratories will report results of both tests when undertaken, these data do not represent the total numbers confirmed by any one method, but do indicate a wider adoption of PCR as a screening tool. Ideally, records in SGSS should be updated when additional diagnostic tests are conducted – for example when a PCR test is followed up with culture. However, the processes behind different LIMS vary, which can lead to inconsistencies with how records are updated. Whilst the overall number of PCR results versus culture results generally align to the proportion of isolates received by the reference laboratory, the lack of a standardized approach makes is challenging to accurately determine the diagnostic test methods used in front-line laboratories.

**Figure 4.**
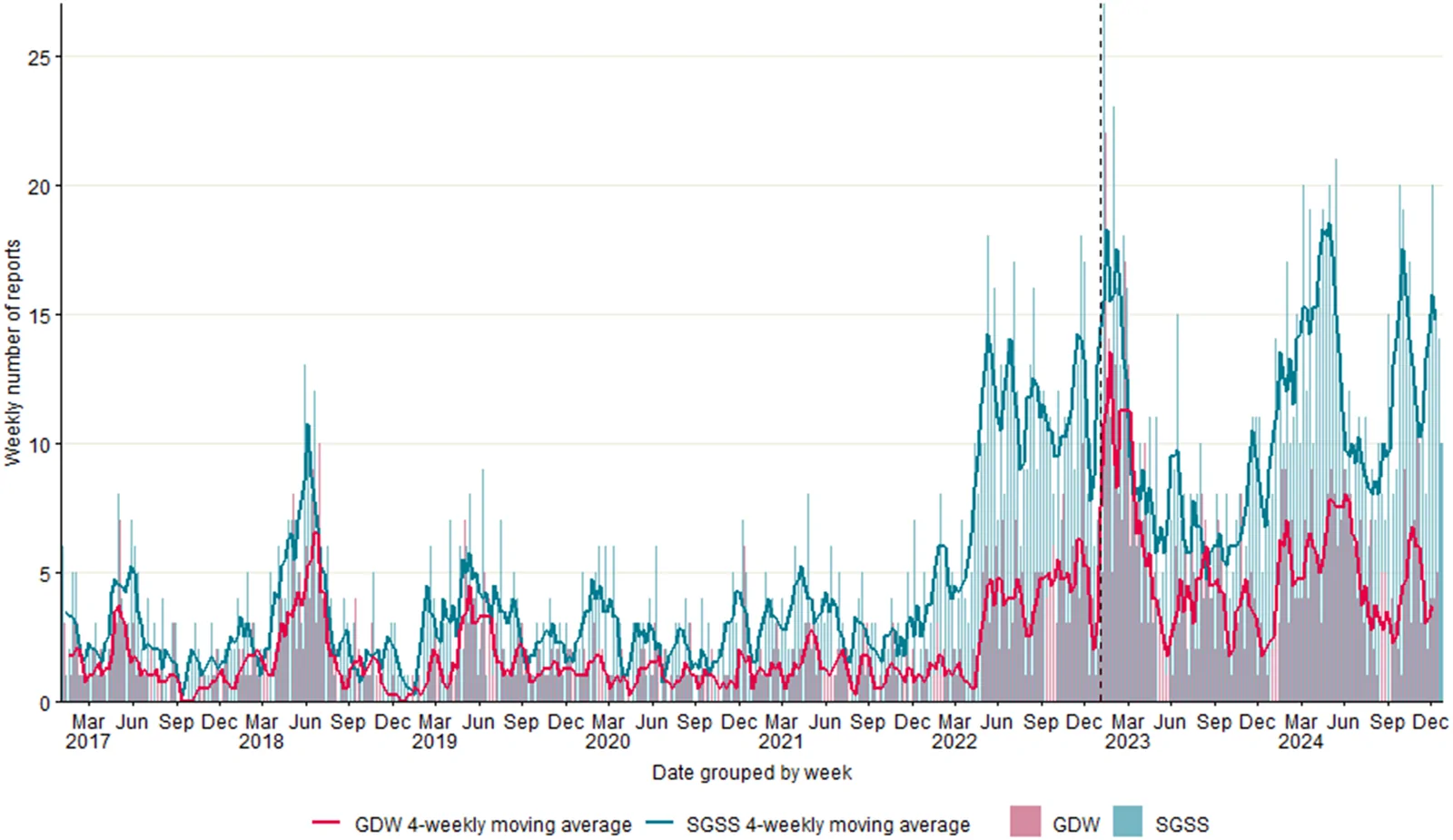
Comparison of SGSS and GDW reporting of *Yersinia*. A four-week moving average is calculated and indicated by the blue (SGSS) and pink (GDW) lines. Cases are grouped by week. Black dotted line in January 2023 indicates when the request for regional laboratories to culture and send to the reference laboratory was published. Figure 4. long description.

Between 2017 and 2024, there were 1073 unique *Yersinia* spp. isolates referred to GBRU from clinical laboratories in England for WGS. This is approximately 46% of all detections of *Yersinia* spp. recorded in SGSS (Table 2, Figure 4, and Supplementary Figure 2). During the five pre-exceedance years (2017–2021) there were 370 (34%, 370/1073, average 74 per year) reported referrals to GBRU, which increased to 703 referrals (66%, 703/1073, average 234 per year) in 2022–2024. The increase in cases reported to SGSS in 2022–2024 was mirrored in GBRU referrals based on historic baseline numbers (Figure 4).

**Table 2.** Comparison of GBRU referrals to SGSS detections per region Table 2. long description.

	2017–2021 (GBRU/SGSS, %)	2022–2024 (GBRU/SGSS, %)	Total (GBRU/SGSS, %)
East Midlands	35	49	44
East of England	55	85	72
London	23	33	29
North East	75	39	47
North West	76	43	49
South East	55	46	49
South West	17	19	19
West Midlands	39	49	46
Yorkshire and Humber	25	49	42
Total	50	44	46

*Note:* There was no region available for five cases in this table. One case in the 2017–2021 period and four cases in the 2022–2024 period.

Values are a percentage of SGSS entries that are referred to the GBRU (GBRU/SGSS (%)). Some percentages may not sum to 100% due to rounding.

The East of England, most often referred to the GBRU, followed by the North West and South East as mirrored with reports to SGSS (Table 2). Despite the change in regional referral guidelines for *Yersinia* spp. to the GBRU in January 2023, there is still a discrepancy in the number of referred isolates compared to the number reported to SGSS. These differences could be attributed to difficulty in obtaining a pure culture of the sample at the local lab, as well as PCR-positive, culture-negative results (non-viable pathogen), variability in sending practices, and lack of engagement with the reference laboratory and good surveillance practice.

In 2021, the North East conducted a pilot study of enhanced testing and referral of *Yersinia* spp. in all patient groups in collaboration with GBRU, which likely accounts for the higher reporting to SGSS and referral of isolates to the GBRU in 2017–2021. Between 2022 and 2024, the North East laboratory was no longer conducting the study, highlighting how singular labs implementing PCR can impact trends.

### Sample types

Matching cases using name and date of birth between SGSS and GDW resulted in 807 cases with a record in SGSS and GDW. Due to insufficient completion of specimen types in GDW, where data matched across both datasets, the specimen reported in SGSS was used due to this variable being more comprehensively completed. There were also bi-directional gaps between the two databases due to variations in reporting procedures.

There was an increase in sterile site detections (Figure 1) and referrals to the reference laboratory (Table 3) between 2017–2021 and 2022–2024, attributed to early 2022. Blood and other sterile site isolations to the GBRU in 2017–2021 accounted for 6% of referrals, which increased to 12% in 2022–2024 (Table 3).

**Table 3.** Referred notifications by sample type Table 3. long description.

Specimen type	2017–2021		2022–2024		Total
	*n*	%	*n*	%	*n*
Blood	15	5	57	11	72
Faeces	261	92	449	86	710
Sputum	1	1	1	1	2
Other sterile sites	2	2	6	0	8
Other non-sterile sites	6	0	2	1	8
Unknown	0	0	7	0	7
Total	285	100	522	100	807

*Note:* Other sterile sites reported included tissue, joint (prosthesis), urine, and peritoneal dialysate.

The table indicates referred sample types between 2017–2021 (pre-exceedance) and 2022–2024 (during/post exceedance) where records were present in SGSS and GDW (*n* = 807) matched on name and date of birth. Sample type recorded in SGSS is used as this is more complete. Some percentages may not sum to 100% due to rounding.

### Species

*Y. enterocolitica* is the dominant species referred to the GBRU by clinical laboratories (89%, 950/1073) with *Y. pseudotuberculosis* typed second most often, but at lower levels (3%, 29/1073) (Supplementary Table 2). The incidence of both remained consistent between the 2017–2021 and 2022–2024 periods, with both species mirroring the global picture (Supplementary Table 2). Of the *Yersinia* spp. that were speciated, the proportion of *Y. pseudotuberculosis* isolated from sterile sites was higher (14/29, 48%; blood = 13, pleural fluid = 1) than *Y. enterocolitica* (55/950, 6%; blood = 54, ascitic fluid = 1). One patient had *Yersinia kristensenii* isolated from cerebrospinal fluid and *Y. alsatica* from blood^‡^.

### Antibiotic susceptibility

Antibiotic susceptibility was tested on 71 (2 *Y. pseudotuberculosis* and 69 *Y. enterocolitica*) isolates from faeces and 92 (14 *Y. pseudotuberculosis* and 78 *Y. enterocolitica*) isolates from blood (Amikacin, Ampicillin, Azithromycin, Chloramphenicol, Ciprofloxacin, Colistin, Gentamicin, Nalidixic Acid, Sulfamethoxazole, Tetracycline, Tigecycline, Trimethoprim, Ertapenem, Cefepime, Cefotaxime, Cefoxitin, Imipenem, Meropenem, Ceftazidime, Temocillin). The distributions of MIC are presented in Supplementary Table 3, with breakpoints (where available) and dilutions tested. The results show that the majority of isolates of *Y. enterocolitica* are resistant to ampicillin; this is as expected, as this species is naturally resistant to ampicillin and first- and second-generation cephalosporins due to intrinsic production of beta-lactamases by this species [43–45]. All except one isolate were sensitive to ciprofloxacin and third-generation cephalosporins; all were sensitive to gentamicin, azithromycin, chloramphenicol, and carbapenems. All *Y. pseudotuberculosis* isolates tested were resistant to colistin, but remained sensitive to beta-lactams, carbapenems, ciprofloxacin, chloramphenicol, gentamicin, azithromycin, and trimethoprim [43–45].

### Travel-associated detections

Travel data is often poorly captured on laboratory forms, with only 8% (85/1073) reported a travelling and/or travel location on the laboratory form. Consequently, there are limitations around inferring travel status based on laboratory forms and the following results should be interpreted with caution.

The following detections were reported in individuals who had travelled prior to onset: *Yersinia rohdei* (1/1, 100%), *Yersinia mollaretii* (2/3, 67%), *Yersinia massiliensis* (1/2, 50%), *Y. proxima* (2/21, 10%), *Yersinia frederiksenii* (4/25, 16%), *Y. pseudotuberculosis* (3/29, 10%), *Y. canariae* (1/11, 9%), and *Y. enterocolitica* (71/950, 7%), with travel to Turkey, Spain, India, and Egypt as the most frequently reported travel destinations.

### Enteric survey results for *Yersinia* spp.

In 2023, 93 diagnostic laboratories took part in a survey on laboratory testing practices and reported that 89/93 (96%) laboratories had the capacity to detect *Yersinia.* There were 7/89 (7.9%) laboratories using enteric PCR routinely for detection of *Yersinia* spp. either as a standalone PCR or using a combination of PCR and selective culture. An additional five laboratories have PCR capacity for *Yersinia* but use it more selectively, such as for private patients or rapid PCR only for inpatients and new admissions only (Supplementary Figure 2 and Supplementary Table 4). No laboratories use PCR for detection of *Yersinia* in all patient groups in the South West, North East, Midlands, or East of England.

Of the laboratories using enteric PCR, 7/12 (58%) would report PCR-only detections of *Yersinia* spp. to SGSS, although this ranges from 75% in the South East and London to 0% in Yorkshire and Humber. As only one laboratory reported using PCR in isolation without culture, it is likely the majority of cases would still be reported to SGSS as a culture-positive.

However, only 9/89 (10%) laboratories test all community stool specimens for *Yersinia* (PCR only *n* = 2, culture only *n* = 2, PCR and culture *n* = 3), and this was across five regions (Yorkshire and Humber *n* = 1, East of England *n* = 2, South East *n* = 2, London *n* = 2, and North West *n* = 2). No laboratories in the South West, North East, or Midlands tested all community groups. Of laboratories not testing all specimens, two reported testing all care home, GP, recent admissions and paediatric groups, whilst the 77/89 (87%) only test where specifically requested or clinical details indicate to (as recommended by the SMI). The two laboratories reporting ‘other groups’ reported testing in those under 16 years old, with abdominal pain and as part of an expanded test panel for patients with private healthcare.

## Discussion

Historical surveillance data from the UK, current surveillance data from other countries in Europe and elsewhere, and preliminary data from the third Infections Intestinal Disease Study funded by the Food Standards Agency in the UK, all provide evidence that the incidence of *Y. enterocolitica* is similar to the non-typhoidal *Salmonella* (NTS) species [10, 24, 46]. Both are GI pathogens prevalent in the animal reservoir, have a wide host range and are transmitted to humans via contaminated food and direct or indirect contact with animals or their environment. The low recorded notifications of *Y. enterocolitica* in the UK compared to NTS have been linked to the diagnostic algorithms that specify tests for *Y. enterocolitica* should only be deployed in certain clinical scenarios. As a result, community cases of yersiniosis with GI symptoms have been taking place under the surveillance radar in the UK for decades. Moreover, it is highly likely that hospitalized cases with more severe symptoms were also missed, as clinical details are poorly captured on laboratory submission forms.

The implementation of molecular methods facilitating testing of all faecal specimens submitted to microbiology laboratories for detection of GI pathogens has greatly improved case ascertainment **for* Y. enterocolitica* in England [47]. This change to the diagnostic algorithm is the most likely explanation for the increase in notifications of yersiniosis made to UKHSA since 2022. Common PCR panels used for *Yersinia* detection are restricted to *Y. enterocolitica*, likely contributing to the overrepresentation of *Y. enterocolitica* relative to *Y. pseudotuberculosis* and therefore reducing resolution of the true prevalence of *Yersinia* spp. detected from clinical investigations. The increase in notifications was highest in the South East and North West regions where the implementation of faecal molecular testing was most widespread (UKHSA in-house data). In contrast to other countries in Europe, where the highest proportion of cases are in children, the majority of yersiniosis notifications in England were in adults, with most infections occurring in the over 65 age group [10]. Yersiniosis in the elderly population may be related to waning immunity and/or a higher proportion of underlying health conditions in this vulnerable group as well as enhanced testing on those with chronic conditions detecting asymptomatic cases.

The majority of isolates were *Y. enterocolitica*, and of the other *Yersinia* species detected, *Y. pseudotuberculosis* was the only other species known to be pathogenic to humans. Most cultures were isolated from faecal specimens, although the increase in isolations from extraintestinal sites provides further evidence of the invasive nature of this pathogen and the potential to cause more severe sequelae following primary GI infection. Recent studies have suggested that types of *Y. enterocolitica* previously thought to be non-pathogenic can cause symptoms of GI infection, and enhanced clinical and epidemiological studies would need to be conducted to explore whether this may apply to other *Yersinia* species as well [48]. Post 2021, more diverse species were reported, most likely due to improvements to the diagnostic algorithm, changes, and developments in resolution of databases and sequencing technologies across the years, as well as the discovery of new species across time [3–7]. The pathogenic potential of *Yersinia* species other than *Y. enterocolitica* and *Y. pseudotuberculosis* remains unclear [49]. *Y. enterocolitica* as a species has varying pathogenicity that current evidence suggests can be defined through biotyping. In the genomic era, WGS speciation and genomic/virulence typing of *Yersinia* paired with enhanced clinical information could provide further evidence of pathogenic and non-pathogenic *Y. enterocolitica* and other *Yersinia* species.

However, this study provides evidence that *Yersinia* species other than *Y. enterocolitica* and *Y. pseudotuberculosis* have been detected from individuals with symptoms consistent with gastroenteritis and/or other symptoms consistent with infection; however, further analysis is required to attribute the symptoms to the clinical infection and genomic features. *Y. kristensenii* and *Y. alsatica* were detected in sterile sites from the same case, and whilst this does not establish causation, it indicates that species other than *Y. enterocolitica* and *Y. pseudotuberculosis* may occasionally be detected in an invasive isolation and warrants further investigation. The systematic collection of enhanced surveillance data, including clinical outcomes and onset dates, would begin to resolve this gap in knowledge. Enhanced surveillance data would also provide more detailed epidemiological understanding of *Yersinia*, for example by enabling us to determine the true incidence of travel-associated yersiniosis and compare domestic and imported detections.

We found that less than 50% of cases notified at the local level were referred to GBRU for confirmation and typing. The reasons for this are unclear but may be partly due to a proportion of the faecal specimens being positive by PCR only, either because the laboratory did not attempt culture or culture was negative, and there was no isolate to refer. When interpreting data from the enteric survey, it is important to recognize that regions and laboratories might have developed detection methods and begun more widespread PCR roll-out since the study. *Yersinia* species grow as small translucent colonies and can be difficult to identify on semi-selective media. Furthermore, generally PCR is more sensitive than culture [50, 51]. Limited referral may also be due to the perception that yersinosis is not a clinical or public health priority. This is a misconception as clinically *Y. pseudotuberculosis* and certain types of *Y. enterocolitica* are associated with severe abdominal pain and secondary sequalae, including appendicitis, mesenteric lymphadenitis, terminal ileitis or reactive arthritis and sepsis [15]. From a public health perspective, the burden of community-acquired diarrhoea is likely to be much higher than the notification numbers suggest, and the zoonotic and foodborne nature of the pathogen means that large, foodborne outbreaks are possible [52].

Gastroenteritis caused by *Yersinia* is usually self-limiting; however, antimicrobial treatment is recommended in patients with severe or prolonged gastroenteritis, the immunocompromised and those with complications such as sepsis. The antimicrobial susceptibilities and MIC distributions suggest a vast majority of strains tested were sensitive to routinely used therapeutic agents such as fluoroquinolones, aminoglycosides, chloramphenicol, carbapenems, and azithromycin. *Y. enterocolitica* is inherently resistant to beta-lactams and first- and second-generation cephalosporins due to chromosomal beta-lactamases. *Y. pseudotuberculosis* is considered to be resistant to colistin due to the presence of *cpx*-signalling genes; our results indicated these expected findings [43–45]. It is important to note that the number of strains tested in this study was relatively low and therefore further validation work is required to confirm the resistance trends presented and how the trends represent the wider *Yersinia* spp. population. Isolates should be tested appropriately when treatment is indicated.

WGS and determination of strain relatedness is vital in quickly identifying outbreaks [53]. During the exceedance period in 2022, regional laboratories were not routinely referring to GBRU; in addition, the epidemiological significance of a given single nucleotide polymorphism clustering threshold for *Yersinia* in England is currently undetermined. Therefore, the lack of genomic surveillance of *Yersinia* and food/environmental exposure questionnaires not being routinely administered at this time point resulted in an inability to rapidly identify outbreaks and conduct public health intervention. These historic limitations are being addressed within UKHSA.

The developments in surveillance across this study period in England, including PCR introduction and mandatory referrals, add complexity to the interpretation of trends. Whilst the increase in *Yersinia* detections is likely reflective of true incidence, notifications are also impacted by improved ascertainment in diagnostic laboratories; therefore, it is important that trends are interpreted with caution. Other limitations are recognized in this study that result from using a live database, which often involves retrospective reporting. Consequently, case numbers reported here may differ from those reported elsewhere [10, 42]. Furthermore, there can be discrepancies in variables reported between two data sources, such as region or name spelling, which consequently impact data matching. Finally, bidirectional gaps between data sources due to non-standardized methods of reporting in regional labs, as well as new records created rather than updating old records post-WGS can result in lower resolution of novel species reporting due to de-duplication methods. Therefore, all isolates should be referred to the reference laboratory for high-resolution genomic typing. This would result in the data from the reference laboratory being representative of all yersiniosis cases, not ~40%–50%. A comprehensive analysis of WGS data from genomes of *Yersinia* would strengthen the current understanding of virulence marker distribution across *Yersinia* spp.

Under the Health Protection (Notification) Regulations 2010 (HPNR), all registered medical practitioners are required to report notifiable diseases to the Proper Officer of the local authority (usually a UKHSA regional team). Since 6 April 2025, diagnoses of *Yersinia* species must be notified to UKHSA [29], and this may encourage more widespread implementation of PCR and confirmation by culture, improve rates of referral of isolates for typing, and highlight the clinical and public health significance of yersiniosis. Referral of isolates to GBRU for WGS is important and recommended, as such data provide robust speciation, serotyping, and typing for outbreak detection as well as identification of virulence markers for assessment of pathogenicity. We recommend systematic, real-time enhanced surveillance data collection to integrate the genomic data with good quality clinical and epidemiological data in order to provide a comprehensive perspective of yersiniosis epidemiology in England.

## Supporting information

10.1017/S0950268826102167.sm001 Rodwell et al. supplementary material Rodwell et al. supplementary material

## Data Availability

The FASTQ reads from all sequences in this study can be found at the UKHSA Pathogens (*Yersinia*) BioProject at the National Center for Biotechnology Information (PRJNA481015).

## Supplementary material

The supplementary material for this article can be found at http://doi.org/10.1017/S0950268826102167.

## Long descriptions

### Figure 1. Long description

Number of detections on the y-axis and the year on the x-axis. Detections are grouped monthly by sample type. Samples from typically sterile sites are presented in teal, from non-sterile sites in light blue and unknown sample type in grey/blue with a 12 month rolling average presented in pink. Detections from typically sterile sites peaked in 2022 and 2023 and detection from non-steile sites increased rapidly in 2022 and remained higher than 2017–2021.

Navigate back to Figure 1..

### Figure 2. Long description

Number of detections on the y-axis and the week of the year the specimen was taken on the x-axis. Detections are presented in four time frames, the 5 year range between 2017 and 2021 with the median as a light blue dotted line and the range shaded pink; 2022 in coral; 2023 in light blue; and 2024 in dark blue. The figure shows an increase in detections in 2022, 2023 and 2024 when compared to the 5 year range.

Navigate back to Figure 2..

### Figure 4. Long description

Weekly number of reports on the y-axis and month and year on the x-axis. Local diagnostic laboratory (SGSS) reports are presented as blue bars, with a 4-week rolling average overlayed as a blue line. Reference laboratory reports (GDW) are presented as pink bars, with a 4-week rolling average overlayed as a pink line. A vertical dotted line in January 2023 indicates when recommendations for isolate referal to the reference laboratory for all *Yersinia* spp. was published.

Navigate back to Figure 4..

### Table 1. Long description

The table is divided into three main columns for time periods: 2017 to 2021, 2022 to 2024, and Total. Each period includes sub-columns for count (n), percentage (%), and detections per 100,000 (D p H).

Age group section:

* 0 to 4: Total n = 161, 7%, D p H 0.64.

* 5 to 14: Total n = 158, 7%, D p H 0.29.

* 15 to 24: Total n = 179, 8%, D p H 0.33.

* 25 to 44: Total n = 441, 19%, D p H 0.36.

* 45 to 64: Total n = 575, 24%, D p H 0.50.

* Over 65: Total n = 836, 36%, D p H 1.00. This group shows the highest increase in D p H from 0.43 in 2017 to 2021 to 1.91 in 2022 to 2024.

* Total: n = 2350, 100%.

Region section:

* East Midlands: Total n = 63, 3%, D p H 0.16.

* East of England: Total n = 85, 4%, D p H 0.17.

* London: Total n = 143, 6%, D p H 0.20.

* North East: Total n = 91, 4%, D p H 0.43.

* North West: Total n = 335, 14%, D p H 0.56.

* South East: Total n = 1249, 53%, D p H 1.68. This region accounts for over half of all notifications.

* South West: Total n = 188, 8%, D p H 0.41.

* West Midlands: Total n = 74, 3%, D p H 0.16.

* Yorkshire and The Humber: Total n = 123, 5%, D p H 0.28.

* Total: n = 2351, 100%.

Navigate back to Table 1..

### Table 2. Long description

The table consists of four columns and eleven rows. The columns are: Region, 2017 to 2021 (G B R U forward slash S G S S, percent), 2022 to 2024 (G B R U forward slash S G S S, percent), and Total (G B R U forward slash S G S S, percent).

* East Midlands: 35, 49, 44.

* East of England: 55, 85, 72.

* London: 23, 33, 29.

* North East: 75, 39, 47.

* North West: 76, 43, 49.

* South East: 55, 46, 49.

* South West: 17, 19, 19.

* West Midlands: 39, 49, 46.

* Yorkshire and Humber: 25, 49, 42.

* Total: 50, 44, 46.

Note: Values represent the percentage of S G S S entries referred to the G B R U. Five cases had no region available.

Navigate back to Table 2..

### Table 3. Long description

The table consists of six columns: Specimen type, 2017 to 2021 (n and percentage), 2022 to 2024 (n and percentage), and Total n.

* Blood: 15 (5 percent) in 2017 to 2021; 57 (11 percent) in 2022 to 2024; Total 72.

* Faeces: 261 (92 percent) in 2017 to 2021; 449 (86 percent) in 2022 to 2024; Total 710.

* Sputum: 1 (1 percent) in 2017 to 2021; 1 (1 percent) in 2022 to 2024; Total 2.

* Other sterile sites: 2 (2 percent) in 2017 to 2021; 6 (0 percent) in 2022 to 2024; Total 8.

* Other non-sterile sites: 6 (0 percent) in 2017 to 2021; 2 (1 percent) in 2022 to 2024; Total 8.

* Unknown: 0 (0 percent) in 2017 to 2021; 7 (0 percent) in 2022 to 2024; Total 7.

* Total: 285 (100 percent) in 2017 to 2021; 522 (100 percent) in 2022 to 2024; Grand Total 807.

Note: Other sterile sites include tissue, joint prosthesis, urine, and peritoneal dialysate. Data is matched from S G S S and G D W records.

Navigate back to Table 3..
