# Supplementary material for: Public health surveillance of Yersinia species in England, 2017–2024
Source: Epidemiol Infect. 2026 Oct 14;154:e132. doi: 10.1017/S0950268826102167 (PMC13749194; doi:10.1017/S0950268826102167)
Supplement: Supplementary file 1 [file S0950268826102167sup001.docx]

Supplementary Information

Supplementary Figure 1 – Test method distribution reported in the second generation surveillance system

Supplementary Figure 2 – Total regional gastrointestinal bacteria reference unit vs the second generation surveillance system referral percentages (%). Laboratories conducting PCR at the time of the enteric survey in 2023 indicated with red filled circles.

Supplementary Table 1 – Infections per 100000 per year.

| Year | Count (n) | Infection per 100000 |
| --- | --- | --- |
| 2017 | 120 | 0.2 |
| 2018 | 179 | 0.3 |
| 2019 | 152 | 0.3 |
| 2020 | 135 | 0.2 |
| 2021 | 154 | 0.3 |
| 2022 | 487 | 0.9 |
| 2023 | 454 | 0.8 |
| 2024 | 670 | 1.2 |

Supplementary Table 2: Speciation of *Yersinia* spp. at the gastrointestinal bacteria reference unit. Table indicates referred sample types and species typed between 2017-2021 (pre-exceedance) and 2022-2024 (during/post exceedance)

|  |  | **2017-2021** | | **2022-2024** | | **Total** | |
| --- | --- | --- | --- | --- | --- | --- | --- |
|  |  | **n** | **%** | **n** | **%** | **n** | **%** |
| **Species** | ***Yersinia alsatica*** | 0 | 0 | 10 | 2 | 10 | 1 |
|  | ***Yersinia artesiana*** | 0 | 0 | 2 | 0 | 2 | 0 |
|  | ***Yersinia bercovieri*** | 2 | 0 | 1 | 0 | 3 | 0 |
|  | ***Yersinia canariae*** | 0 | 0 | 11 | 2 | 11 | 1 |
|  | ***Yersinia enterocolitica*** | 321 | 87 | 629 | 89 | 950 | 89 |
|  | ***Yersinia frederiksenii*** | 19 | 5 | 6 | 1 | 25 | 2 |
|  | ***Yersinia intermedia*** | 5 | 1 | 2 | 0 | 7 | 1 |
|  | ***Yersinia kristensenii*** | 0 | 0 | 2 | 0 | 2 | 0 |
|  | ***Yersinia massiliensis*** | 2 | 0 | 0 | 0 | 2 | 0 |
|  | ***Yersinia mollaretii*** | 1 | 0 | 2 | 0 | 3 | 0 |
|  | ***Yersinia proxima*** | 8 | 0 | 13 | 2 | 21 | 2 |
|  | ***Yersinia pseudotuberculosis*** | 9 | 2 | 20 | 3 | 29 | 3 |
|  | ***Yersinia rohdei*** | 1 | 0 | 0 | 0 | 1 | 0 |
|  | ***Yersinia sp.*** | 2 | 0 | 1 | 0 | 3 | 0 |
|  | ***Yersinia vastinensis*** | 0 | 0 | 4 | 1 | 4 | 0 |
|  | **Total** | **370** | **100** | **703** | **100** | **1073** | **100** |

Supplementary Table 3: MIC of Yersinia enterocolitica and Yersinia pseudotuberculosis. YE = Yersinia enterocolitica, YP = Yersinia pseudotuberculosis. The numbers in the cells correlate to the number of isolates. Shaded red cells indicate where the EUCAST Enterobacterales breakpoints indicate resistance. Black lines indicate the range of MIC tested for that antibiotic.

| **Species** | **Antibiotic** | **0.015** | **0.03** | **0.06** | **0.12** | **0.25** | **0.5** | **1** | **2** | **4** | **8** | **16** | **32** | **64** | **128** | **256** | **512** | **>512** |
| --- | --- | --- | --- | --- | --- | --- | --- | --- | --- | --- | --- | --- | --- | --- | --- | --- | --- | --- |
| **YE** | Amikacin |  |  |  |  |  |  |  |  | 145^i^ | 2 |  |  |  |  |  |  |  |
| **YE** | Ampicillin |  |  |  |  |  |  | 4^g^ |  |  | 12 | 11 | 79  41^l^ |  |  |  |  |  |
| **YE** | Azithromycin* |  |  |  |  |  |  |  | 4^h^ | 111 | 32 |  |  |  |  |  |  |  |
| **YE** | Chloramphenicol* |  |  |  |  |  |  |  |  |  | 146^j^ |  |  | 1^m^ |  |  |  |  |
| **YE** | Ciprofloxacin | 26^a^ | 110 | 7 | 2 |  | 1 | 1 |  |  |  |  |  |  |  |  |  |  |
| **YE** | Colistin |  |  |  |  |  |  | 125^g^ | 15 |  | 1 | 6^k^ |  |  |  |  |  |  |
| **YE** | Gentamicin |  |  |  |  |  | 124^f^ | 22 | 1 |  |  |  |  |  |  |  |  |  |
| **YE** | Nalidixic Acid |  |  |  |  |  |  |  |  | 142^i^ | 3 |  |  | 2^m^ |  |  |  |  |
| **YE** | Sulfamethoxazole** |  |  |  |  |  |  |  |  |  | 95 ^j^ | 37 | 7 | 2 |  | 2 | 1 | 3 |
| **YE** | Tetracycline* |  |  |  |  |  |  |  | 112^h^ | 32 | 1 |  | 2^l^ |  |  |  |  |  |
| **YE** | Tigecycline**** |  |  |  |  | 133^e^ | 13 | 1 |  |  |  |  |  |  |  |  |  |  |
| **YE** | Trimethoprim*** |  |  |  |  | 1^e^ | 11 | 51 | 75 | 7 |  | 2^k^ |  |  |  |  |  |  |
| **YE** | Ertapenem^†^ | 14^a^ | 99 | 31 | 2 |  |  |  |  |  |  |  |  |  |  |  |  |  |
| **YE** | Cefepime^†^ |  |  | 89^c^ | 56 |  |  | 1 |  |  |  |  |  |  |  |  |  |  |
| **YE** | Cefotaxime^†^^ |  |  |  |  | 116^e^ | 16 | 5 | 7 |  | 1 |  |  |  |  |  |  |  |
| **YE** | Cefoxitin^†**^ |  |  |  |  |  |  | 3 | 7 | 44 | 14 | 48 | 27 | 3 |  |  |  |  |
| **YE** | Imipenem^†^ |  |  |  | 1^d^ | 89 | 55 | 1 |  |  |  |  |  |  |  |  |  |  |
| **YE** | Meropenem^†^ |  | 104^b^ | 42 |  |  |  |  |  |  |  |  |  |  |  |  |  |  |
| **YE** | Ceftazidime^†^ |  |  |  |  | 63^e^ | 55 | 26 | 1 |  |  |  | 1 |  |  |  |  |  |
| **YE** | Temocillin^†*****^ |  |  |  |  |  | 1^f^ | 1 | 26 | 73 | 31 | 12 | 1 | 1 |  |  |  |  |
| Species | Antibiotic | 0.015 | 0.03 | 0.06 | 0.12 | 0.25 | 0.5 | 1 | 2 | 4 | 8 | 16 | 32 | 64 | 128 | 256 | 512 | >512 |
| **YP** | Amikacin |  |  |  |  |  |  |  |  | 16^i^ |  |  |  |  |  |  |  |  |
| **YP** | Ampicillin |  |  |  |  |  |  | 16^g^ |  |  |  |  |  |  |  |  |  |  |
| **YP** | Azithromycin* |  |  |  |  |  |  |  |  | 7 | 9 |  |  |  |  |  |  |  |
| **YP** | Chloramphenicol* |  |  |  |  |  |  |  |  |  | 15^j^ | 1 |  |  |  |  |  |  |
| **YP** | Ciprofloxacin |  | 7 | 9 |  |  |  |  |  |  |  |  |  |  |  |  |  |  |
| **YP** | Colistin |  |  |  |  |  |  |  |  |  | 1 | 15^k^ |  |  |  |  |  |  |
| **YP** | Gentamicin |  |  |  |  |  | 16^f^ |  |  |  |  |  |  |  |  |  |  |  |
| **YP** | Nalidixic Acid |  |  |  |  |  |  |  |  | 16^i^ |  |  |  |  |  |  |  |  |
| **YP** | Sulfamethoxazole** |  |  |  |  |  |  |  |  |  | 15^j^ | 1 |  |  |  |  |  |  |
| **YP** | Tetracycline* |  |  |  |  |  |  |  | 16^h^ |  |  |  |  |  |  |  |  |  |
| **YP** | Tigecycline**** |  |  |  |  | 16^e^ |  |  |  |  |  |  |  |  |  |  |  |  |
| **YP** | Trimethoprim*** |  |  |  |  | 6^e^ | 9 | 1 |  |  |  |  |  |  |  |  |  |  |
| **YP** | Ertapenem | 16^a^ |  |  |  |  |  |  |  |  |  |  |  |  |  |  |  |  |
| **YP** | Cefepime |  |  | 15^c^ | 1 |  |  |  |  |  |  |  |  |  |  |  |  |  |
| **YP** | Cefotaxime |  |  |  |  | 16^e^ |  |  |  |  |  |  |  |  |  |  |  |  |
| **YP** | Cefoxitin** |  |  |  |  |  |  |  | 16 |  |  |  |  |  |  |  |  |  |
| **YP** | Imipenem |  |  |  |  | 2 | 14 |  |  |  |  |  |  |  |  |  |  |  |
| **YP** | Meropenem |  | 16^b^ |  |  |  |  |  |  |  |  |  |  |  |  |  |  |  |
| **YP** | Ceftazidime |  |  |  |  | 16^e^ |  |  |  |  |  |  |  |  |  |  |  |  |
| **YP** | Temocillin***** |  |  |  |  |  |  |  | 16 |  |  |  |  |  |  |  |  |  |

a=MIC <=0.015, b=MIC <=0.03, c=MIC <=0.06, d=MIC <=0.12, e=MIC <=0.25, f=MIC <=0.5, g=MIC <=1, h=MIC <=2, i=MIC <=4, j=MIC <=8, k=MIC >16 , l=MIC>32, m=MIC>64. *Breakpoint uncertain - information from notes in EUCAST table,**No EUCAST breakpoint available for Enterobacterales,***Based on breakpoint for uncomplicated UTI,****Based on information available for only E. coli and C. koseri,*****Only meant for UTIs for E. coli, Klebsiella and P. mirabilis. ^†^Indicates one isolate was not screened on this plate (denominator = 146). ^Indicates one isolate had an invalid result

Supplementary Table 4: Further PCR detection methods in laboratories.

| Test Method | NHS District General | NHS Teaching | Private | Joint Venture | Total |
| --- | --- | --- | --- | --- | --- |
| PCR (with/without culture) | 4 | 1 | 1 | 1 (General-Teaching hospital) | 7 |
| PCR capacity (selective use) | 3 | 1 | 0 | 1 (National Health Service-Private healthcare) | 5 |
